# The Cost of Medications at a Student-Run Free Clinic in New Haven, Connecticut, 2021–2023

**DOI:** 10.5888/pcd21.230277

**Published:** 2024-05-16

**Authors:** Claudia See, Krupa Hegde, Lucy Reid, Ryan Shi, Ragini Luthra, Weilai Dong, Viola Lee, Angela Kang-Giaimo

**Affiliations:** 1 Yale School of Medicine, New Haven, Connecticut; 2 Yale University, New Haven, Connecticut; 3 Yale School of Public Health, New Haven, Connecticut; 4 Department of Internal Medicine, Yale New Haven Hospital, Yale School of Medicine, New Haven, Connecticut

## Abstract

**Introduction:**

Haven is a student-run free clinic in New Haven, Connecticut, that serves more than 500 patients annually. Haven’s pharmacy department helps patients obtain medications by providing discount coupons or medications from the clinic’s in-house pharmacy, directly paying for medications at local pharmacies, and delivering medications to patients’ homes. This study aimed to identify prescriptions that have the highest cost among Haven patients.

**Methods:**

Our sample consisted of all Haven patients who attended the clinic from March 2021 through March 2023. Patients were eligible to be seen at Haven if they were aged 18 to 65 years, lacked health insurance, and lived in New Haven. We determined the lowest cost of each medication prescribed to Haven patients by comparing prices among local pharmacies after applying a GoodRx discount. We defined expensive medication as more than $20 per prescription. We excluded medical supplies.

**Results:**

Of the 594 Haven patients in our sample, 64% (n = 378) required financial assistance and 22% (n = 129) were prescribed at least 1 expensive medication. Among 129 patients prescribed an expensive medication, the mean (SD) age was 45.0 (12.3) years; 65% were women, and 87% were Hispanic or Latino. Median (IQR) household annual income was $14,400 [$0–$24,000]. We identified 246 expensive medications; the median (IQR) price per prescription was $31.43 ($24.00–$52.02). The most frequently prescribed expensive medications were fluticasone propionate/salmeterol (accounting for 6% of all expensive medications), medroxyprogesterone acetate (6%), albuterol sulfate (5%), and rosuvastatin (5%).

**Conclusion:**

The average Haven patient has an income well below the federal poverty level, and many have chronic cardiovascular and respiratory conditions that require expensive medications. Future research should work toward making medications universally affordable.

**Summary**

**What is already known on this topic?**

People who lack health insurance also often lack access to medical services and cannot afford prescription medications. Inability to pay for prescription medications can lead to medication nonadherence.

**What is added by this report?**

Among patients at a free clinic, 64% required financial assistance to obtain prescribed medications, and 22% were prescribed an expensive medication, defined as medication costing more than $20.

**What are the implications for public health practice?**

Prescription assistance programs, discount coupons, transportation to pharmacies, and home delivery are ways to make medications more affordable. Student volunteer patient navigators demonstrate the positive effect of student-run clinics on improving access to medications among low-income groups.

## Introduction

Solutions are needed to address the financial cost of medications among people without health insurance. Lack of health insurance is a cause of medication nonadherence and a barrier to improved health. Haven Free Clinic is a student-run primary care clinic that serves more than 500 unique patients annually in 20 departments ranging from pharmacy to social services. Haven’s catchment area is the greater New Haven area in Connecticut. The Haven pharmacy department assists patients in obtaining affordable medication by providing discount coupons, distributing over-the-counter medications from Haven’s basic in-house pharmacy, paying for medication at local pharmacies, and delivering medications to patients’ homes. 

Medication nonadherence is common among patients with low socioeconomic status (1). In 2022, adults without health insurance were more than 2 times as likely as adults with private health insurance to report delaying or not getting a medication due to cost (12.3% vs 5.4%) (2). In 2019, 12.5% of adults aged 19 to 64 years in New Haven had no health insurance (Mark Abraham, executive director, DataHaven, email communication, January 2024), slightly lower than the national rate of 12.9% (2). This comparatively lower rate was in part due to the Affordable Care Act (ACA) (3), which expanded Medicaid insurance for people with low incomes (<138% of the federal poverty level [FPL]) in 31 states, including Connecticut. Among New Haven residents covered by health insurance as a result of the ACA, 77% are members of racial and ethnic minority populations, 73% have no education beyond high school, and 64% live in working families (4). In New Haven in 2019, 26% of the Latino population, 8% of the Black population, and 6% of the non-Hispanic White population had no health insurance (5). New Haven is a town of 134,023 residents (5) and approximately 21 neighborhoods (6); the median income ranges from $31,250 to $87,384 (7). To date, no studies have described the medications and their associated medical conditions that impose the greatest financial burden on people without health insurance. This study investigates this question by using data from the single-center Haven database.

## Methods

This study is a retrospective review of the Haven pharmacy departments’ internal medication database and patient electronic medical records. We included data on all patients seeking care at Haven’s weekly Saturday clinic during a 2-year period, from March 6, 2021, to March 4, 2023. People are eligible to receive care at Haven if they are aged 18 to 65 years, have no health insurance, and live in New Haven County. Patients who have diabetes and require insulin, have HIV/AIDs, need prenatal care, or are receiving active chemotherapy are referred to other health centers and thus were not included in our analyses. Patients who opted out of research were also excluded from our analyses. 

### The Haven clinic

A student-run clinic is a health care delivery program in which students take primary responsibility for the logistics and operational management of services under the supervision of faculty advisors. Frequently, a student-run clinic serves low-income patients who may not have health insurance, are experiencing homelessness, or are at high risk of inadequate management of serious medical problems ranging from hypertension to substance use disorder and violence. Often, student-run clinics provide free access to various services — including blood pressure screening, vaccinations, medications, and laboratory work — and provide low-cost acute care and chronic case management (8).

The Haven Free Clinic (www.havenfreeclinic.com) is a student-run clinic founded in 2005 that partners with Yale University to provide the New Haven community access to comprehensive, high-quality health care free of charge. Haven is run by a group of students from the Yale School of Medicine, Yale School of Nursing, Yale School of Public Health, Yale Physician Associate Program, and Yale University. All health care services are provided by Yale students, under the supervision of licensed physicians, nurse practitioners, and physician associates from the Yale community.

Haven’s existing patient population is predominantly Latino (90%) and Spanish-speaking (85%). The average age of Haven patients is 35 years. However, the clinic serves a diverse population of patients who cannot afford medical care. Many Haven patients have not received medical care for at least 2 to 3 years before coming to Haven (9).

The Haven pharmacy department is made up of graduate and undergraduate students and is typically run by 4 student codirectors assisted by 20 volunteers. The codirectors maintain the in-house inventory of medications, distribute in-house medications at clinic, coordinate pharmacy donation deliveries from nonprofit organizations, assist in the purchase of over-the-counter and prescribed medications (often via telephone), help patients enroll in prescription assistance programs, and coordinate delivery of medications to patient homes. At the clinic, volunteers assist codirectors to perform these tasks. Their main responsibilities are to help distribute medications and identify the most affordable medication options for patients by using GoodRx coupons. 

### Study population

**Figure 1:**
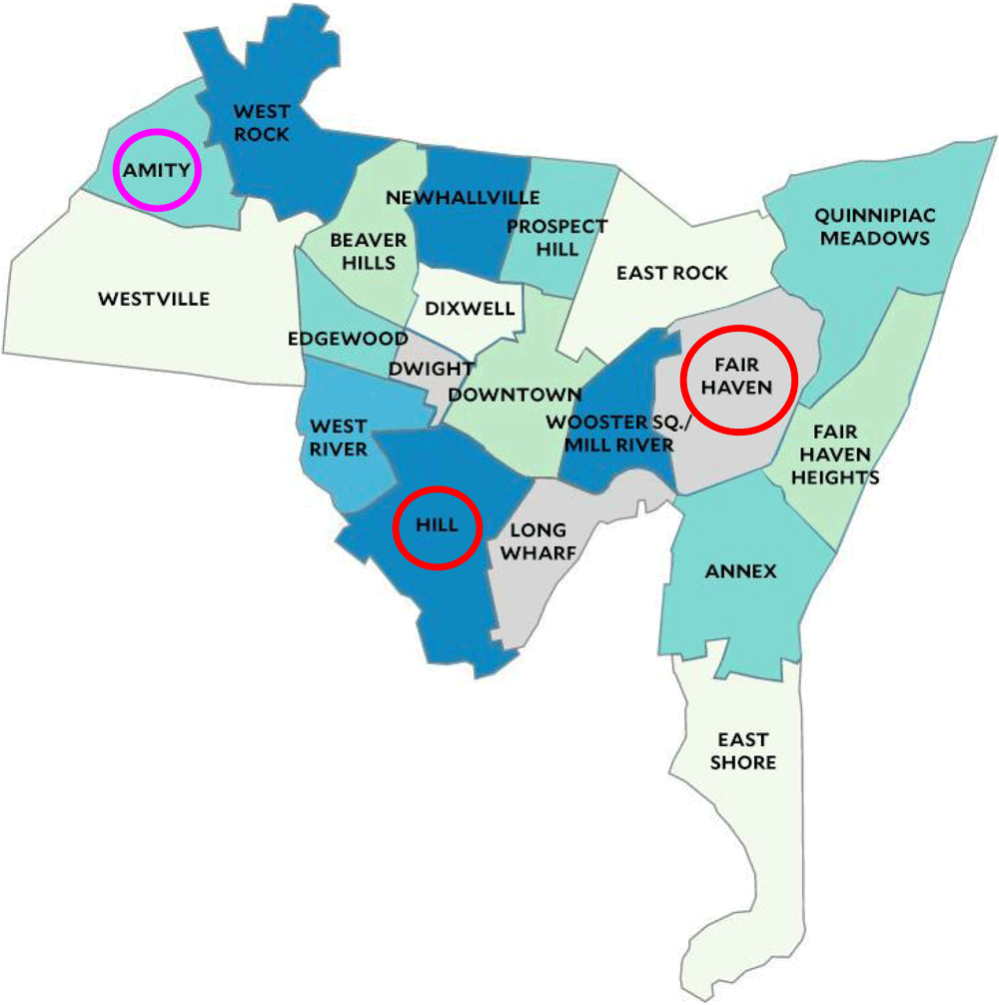
Location of neighborhoods in New Haven, Connecticut. A large proportion (62%) of Haven’s patients live in either the Hill or Fair Haven, 3 to 6 miles from Amity, which has the only retail pharmacy in New Haven that accepts payment over the telephone. Map tiles by Stamen Design, under CC BY 4.0 (15). Data by OpenStreetMap, under ODbL, adapted by the Yale MacMillan Center, which added static neighborhood labels to the image, and used with permission. In addition, 2 red circles were added to highlight low-income neighborhoods (Fair Haven and Hill), and a purple circle was added to highlight a high-income neighborhood (Amity).

The largest proportion (45%) of Haven’s patient population resides in Fair Haven (one of the poorest neighborhoods in New Haven), where the median annual household income was $45,966 in 2021 (10). The Hill, where 17% of the Haven patient population resides, had a median annual household income of $45,416 in 2021 (11). For comparison, in 2021, the median annual US household income was $70,784 (12), $83,572 in Connecticut (13), and $48,973 in New Haven (14). The unemployment rates in Connecticut and in New Haven in 2021 were both 6% (13, 14). The only retail pharmacy that accepts payment over the telephone in New Haven is located in Amity, which poses transportation problems for patients who live 3 to 6 miles away in Fair Haven and the Hill and lack a mode of transportation (Figure 1). Amity is more affluent than the neighborhoods in which our patients live, with a median income of $81,809 in 2021 (16). Neighborhoods in New Haven such as Fair Haven and the Hill may be “pharmacy deserts” — areas with inadequate access to retail pharmacies that disproportionately affect low-income people (17–19).

### Data collection

We extracted the following demographic and health data for each patient from Haven’s electronic medical records: age (in years), sex (male or female), ethnicity (Hispanic or Latino or not), employment (employed or not employed), annual individual income (calculated as weekly income multiplied by 48 weeks or biweekly income multiplied by 24 weeks), annual household income (annual individual income combined for each patient and their legally married spouse if applicable), neighborhood residence within New Haven (21 choices), *International Classification of Diseases, 10th Revision, Clinical Modification* (20) diagnosis or diagnoses for which each expensive medication was prescribed, and comorbidities (all other medical conditions each patient has, excluding diagnoses). The electronic medical records do not include information on race.

Haven’s pharmacy department maintains a database of all medications prescribed by Haven physicians, regardless of where they are dispensed or distributed. The database includes information on medications for which the pharmacy department made external payments to retail pharmacies or for which patients were given the lowest-priced GoodRx coupon to purchase the medication on their own. GoodRx is a free mobile app and website that finds the lowest prescription prices in a person’s neighborhood (21). We excluded medications if 1) data components (eg, price or pharmacy) were not recorded in the database, 2) they were distributed at the clinic rather than paid for at another pharmacy, 3) they were obtained via pharmaceutical company prescription assistance programs, or 4) they were blood pressure cuffs, orthotic braces, or diabetic test supplies. We excluded these medical supplies so that we could focus on medications that directly treat medical conditions. We also excluded insulin because Haven refers patients requiring insulin to other clinics for specialized care.

The medication prices listed in our study are the prices listed on the GoodRx website (www.goodrx.com), which ranges by pharmacy and its location. Haven pharmacy codirectors chose the dispensing pharmacy on the basis of patient preference and the lowest possible price indicated by a GoodRx coupon. We defined expensive medication as medication that cost more than $20 per prescription, regardless of filled amount. We used this $20 cutoff because it is the amount that Haven asks patients to contribute toward prescriptions if they are able. Generally, patients are prescribed enough medication to last them 2 or 3 months, usually until their next visit. Thus, although doses and routes of administration (eg, oral vs topical) may differ, the period of use for each prescription should be similar. We extracted data on the following characteristics of the expensive medications: route of administration (oral, topical, intramuscular, inhalation, otic, suppository, ophthalmic, or vaginal), price per prescription, and type of pharmacy where prescription was filled. We grouped pharmacies into the following categories: retail pharmacy (CVS, Rite Aid, Stop & Shop, Walgreens, and Walmart), mail order pharmacy (Blink Health and Amazon), and hospital pharmacy (Yale New Haven Health Apothecary). We determined the top 10 most commonly prescribed medications and summarized their characteristics (generic name, brand name, number of patients prescribed the medication, the medication count per patient, the range of prices paid, the mean price, the dose, the route of delivery, the type of pharmacy that filled the prescription, and the quantity prescribed). 

We determined that 594 unique patients attended medical visits at Haven during our study period. We used Epic’s Department Appointment Report data from electronic medical records and an internal Haven database stored on Microsoft Teams (Microsoft Corp) to count the total number of unique patients. Medical visits were defined as medical, reproductive health, or tuberculosis therapy appointments.

For data analysis, we calculated percentages for categorical variables and mean (SD) or median (IQR) for continuous variables. We used R version 4.2.3 (R Project for Statistical Computing) to conduct analyses. This study received approval from the Yale Institutional Review Board (no. 2000033657). Data generated during or analyzed for this study are not publicly available because of privacy and ethical restrictions.

## Results

Of 594 unique patients attending Haven for medical visits, 64% (n = 378) required financial assistance from Haven’s pharmacy department and 22% (n = 129) were prescribed an expensive medication. The mean (SD) age of patients receiving an expensive medication was 45.0 (12.3) years; 65% were female and 87% were Hispanic or Latino (Table 1). Forty-five percent lived in Fair Haven, followed by 17% in the Hill and 10% in the Annex. Sixty percent of these patients were employed. Median (IQR) individual and household annual incomes were $11,350 ($0–$19,200) and $14,400 ($0–$24,000), respectively. Of the 263 unique diagnoses associated with the expensive medications, the most common were contraceptive management (9%; n = 24), hyperlipidemia (8%; n = 21), and asthma (8%; n = 21).

**Table 1:** Baseline Characteristics of 129 Patients and Their 263 Medical Diagnoses for Which Expensive Medications^a^ Were Prescribed at Haven Free Clinic, New Haven, Connecticut, March 2021–March 2023^b^

Characteristic	Value
Age, mean (SD), y	45.0 (12.3)
Female, no./total (%)^c^	82/127 (65)
Hispanic or Latino, no./total (%)	112/129 (87)
Employed, no./total (%)^d^	65/108 (60)
Annual income, median (IQR), $^d^	
Individual	11,350 (0–19,200)
Household	14,400 (0–24,000)
Neighborhood residence in New Haven (n = 77), no. (%)^e^	
Fair Haven	35 (45)
Hill	13 (17)
Annex	8 (10)
Fair Haven Heights	5 (6)
Newhallville	4 (5)
East Rock	3 (4)
Edgewood	3 (4)
Quinnipiac Meadows	2 (3)
Prospect Hill	1 (1)
Downtown	1 (1)
Wooster Square/Mill River	1 (1)
Top 10 ICD-10-CM diagnoses**, **no. (%)^f^	
Encounter for contraceptive management, unspecified (Z30.9)	24 (9)
Hyperlipidemia, unspecified (E78.5)	21 (8)
Unspecified asthma, uncomplicated (J45.909)	21 (8)
Primary hypertension (I10)	13 (5)
Gastresophageal reflux disease without esophagitis (K21.9)	7 (3)
Migraine with aura and without status migrainosus, not intractable (G43.109)	6 (2)
H. pylori infection (B96.81)	5 (2)
Migraine without aura, not intractable, without status migrainosus (G43.009)	5 (2)
Other chronic pain (G89.29)	5 (2)
Acne vulgaris (L70.0)	5 (2)
Top 10 comorbidities**, **no. (%)^g^	
Hyperlipidemia	46 (36)
Hypertension	42 (33)
Obesity	25 (19)
Type 2 diabetes^h^	22 (17)
Migraine or headache	20 (16)
Gastresophageal reflux disease	18 (14)
Depression	17 (13)
Hypothyroidism	14 (11)
Latent tuberculosis	14 (11)
Anemia	10 (8)

Abbreviation: ICD-10-CM, *International Classification of Diseases, 10th Revision, Clinical Modification.*

a Expensive medication was defined as >$20 per prescription.

b Data source: patient electronic medical records.

c Denominator excludes 2 patients who identified as transgender female.

d Data on employment and income were available for only 108 patients.

e Excludes 52 patients whose residence could not be confirmed during the study period.

f Denominator is the number of unique diagnoses (n = 263) for which 246 expensive medications were prescribed. A patient’s electronic medical record sometimes listed 2 related ICD-10-CM code diagnoses (20) for which the same medication was prescribed. One example is seasonal allergies (J30.2) and mild intermittent asthma with acute exacerbation (J45.21) for a $29.82 montelukast prescription.

g Denominator is the number of patients (n = 129). Comorbidities are all medical conditions other than the diagnosis for the condition that called for a prescription for an expensive medication.

h Patients who had diabetes and required insulin were excluded from analysis.

The 129 patients were prescribed 113 unique expensive medications encompassing 246 total prescriptions for 263 unique medical diagnoses, totaling $10,967.78 in costs and averaging $42.51 per patient per year. The median (IQR) price per prescription (n = 246 prescriptions), regardless of amount filled was $31.43 ($24.00–$52.02) (Table 2). Of the 113 unique expensive medications, most (82%; n = 93) were purchased from retail pharmacies, followed by mail order pharmacy (8%; n = 9) and a combination of retail pharmacy and mail order pharmacy (4%; n = 5).

**Table 2:** Characteristics of 113 Expensive Medications^a^ Prescribed at Haven Free Clinic, New Haven, Connecticut, March 2021–March 2023^b^

Characteristic	Value
Route of administration, no./113 (%)	
Oral	80 (71)
Topical	15 (13)
Intramuscular	7 (6)
Inhalation	6 (5)
Otic	2 (2)
Suppository	1 (1)
Ophthalmic	1 (1)
Vaginal	1 (1)
Price per prescription, regardless of filled amount (n = 246), median (IQR), $^a^	31.43 (24.00–52.02)
Type of pharmacy, no./113 (%)	
Retail	93 (82)
Mail order	9 (8)
Retail and mail order	5 (4)
Retail and hospital	4 (4)
Hospital	1 (1)
Retail, mail order, and hospital	1 (1)
Top 10 most commonly prescribed medications, no./246 (%)^c^	
Fluticasone propionate/salmeterol (AirDuo RespiClick)	14 (6)
Medroxyprogesterone acetate (Depo-Provera)	14 (6)
Albuterol sulfate (Proventil HFA)	12 (5)
Rosuvastatin (Crestor)	12 (5)
Sumatriptan (Imigran, Migraitan)	10 (4)
Estradiol (Estrace)	7 (3)
Atorvastatin (Lipitor)	5 (2)
Tamsulosin (Flomax)	5 (2)
Tretinoin (Altreno, Atralin, Avita, Retin-A)	4 (2)
Duloxetine (Cymbalta, Yentreve)	4 (2)

a Expensive medication was defined as >$20 per prescription.

b Data sources: patient electronic medical records and GoodRx (price per prescription).

c Includes multiple prescriptions of the same 113 unique expensive medications.

**Figure 2:**
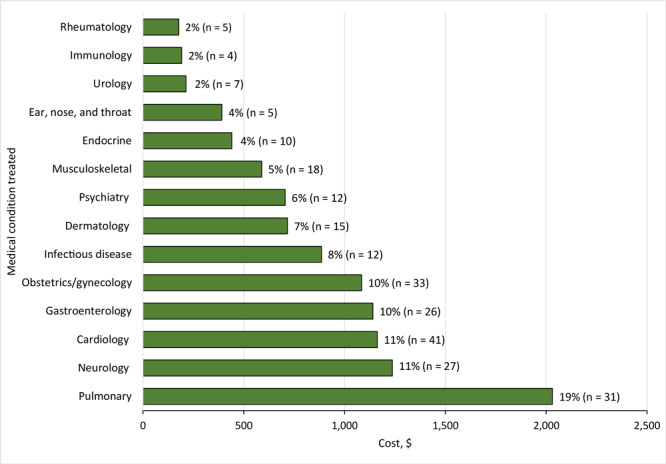
The costs of the 246 expensive prescriptions by type of medical condition treated, Haven Free Clinic, New Haven, Connecticut, March 2021–March 2023. At the top of each bar, the percentage indicates the percentage of the total costs of the expensive medications ($10,967.78) during the study period, and the n’s indicate the number of prescriptions. An expensive medication was defined as one that cost more than $20.

Among the 246 prescribed medications, the most frequently prescribed were fluticasone propionate/salmeterol (6%; n = 14), medroxyprogesterone acetate (6%; n = 14), albuterol sulfate (5%; n = 12), and rosuvastatin (5%; n = 12) (Table 2 and Table 3). The most expensive medications were budesonide ($170.00 for 60 doses of a 90 µg/actuation inhaler), ciprofloxacin–dexamethasone ($146.10 for 7.5 mL of a 0.3%–0.1% otic solution), budesonide–formoterol ($139.43 for 120 doses of an 80/4.5µg actuation inhaler), and fluticasone propionate/salmeterol ($135.00 for 60 doses of a 113–114 µg/actuation inhaler). Three of these 4 most expensive medications were inhalers prescribed for asthma, while the fourth medication — ciprofloxacin–dexamethasone — was prescribed for tympanic rupture. Of the total cost for the 246 prescriptions, 19% of the cost was for a pulmonary condition (n = 31 prescriptions), 11% was for a neurologic condition (n = 27 prescriptions), and another 11% was for a cardiovascular condition (n = 41 prescriptions) (Figure 2). Several patients accounted for substantial medication costs: 1 patient received 3 prescriptions of acetazolamide for idiopathic intracranial hypertension ($239.95 total), another received 3 testosterone gels for hypogonadism ($184.98 total), and 2 patients received 2 prescriptions each of tretinoin for acne vulgaris ($171.94 and $101.03 total).

**Table 3:** The Top 10 Most Common Expensive^a^ Medications Prescribed to Haven Free Clinic Patients, New Haven, Connecticut, March 2021–March 2023^b^

Generic name	Brand name	No. of patients	Medication count per patient	Price paid, range, $^c^	Mean price, $	Dose	Route	Pharmacy type	Quantity
Fluticasone propionate and salmeterol	AirDuo RespiClick	7	2.0	31.38–135.00	80.80	55–14, 113–14, and 232–14 µg per actuation	Inhaler	Retail	0.45 g
Medroxyprogesterone acetate	Depo-Provera	11	1.3	23.70–36.24	30.38	150 mg/mL	Intramuscular	Retail	1 mL
Albuterol sulfate	Proventil HFA	8	1.5	20.01–64.80	29.08	90 µg per actuation	Inhaler	Retail	6.7–8.5 g
Rosuvastatin	Crestor	6	2.0	21.00–33.14	27.97	5–40 mg	Oral	Retail and hospital	60–180 pills
Sumatriptan	Imigran, Migraitan	7	1.4	21.74–77.36	40.75	50–100 mg	Oral	Retail and mail order	30–90 pills
Estradiol	Estrace	6	1.2	22.00–48.42	30.58	0.01%	Topical	Retail	42.5 g
Atorvastatin	Lipitor	5	1.0	21.26–38.00	31.79	10–40 mg	Oral	Retail and mail order	90–180 pills
Tamsulosin	Flomax	3	1.7	22.84–40.31	31.56	0.4–0.8 mg	Oral	Retail and mail order	90–180 pills
Tretinoin	Altreno, Atralin, Avita, Retin-A	2	2.5	44.22–85.97	71.79	0.010%–0.025%	Topical	Retail	45 g
Duloxetine	Cymbalta, Yentreve	4	1.0	20.98–134.40	49.65	20–60 mg	Oral	Retail, mail order, and hospital	60–90 pills

a Expensive medication defined as >$20 per prescription.

b Data sources: patient electronic medical records and GoodRx (price per prescription).

c After applying GoodRx coupon discount at local pharmacies.

**Table:** 

Type of medical condition treated	Total cost, $	Percentage of total costs	Number of prescriptions
Pulmonary	2,030.76	19	31
Neurology	1,236.64	11	27
Cardiology	1,161.96	11	41
Gastroenterology	1,140.94	10	26
Obstetrics and gynecology	1,084.30	10	33
Infectious disease	885.34	8	12
Dermatology	716.62	7	15
Psychiatry	705.80	6	12
Musculoskeletal	589.44	5	18
Endocrine	440.56	4	10
Ear, nose, and throat	391.70	4	5
Urology	213.55	2	7
Immunology	192.32	2	4
Rheumatology	177.85	2	5
Total	10,967.78	100	246

## Discussion

Our study is among the first to describe the financial burden of prescription medications among people without health insurance at a student-run free clinic. Our patient population lives well below the federal poverty level: the median individual annual income was $11,350 and the median annual household income was $14,400, whereas the Connecticut federal poverty level thresholds in 2023 were $14,580, $19,720, and $24,860 for a 1-, 2-, and 3-member household, respectively (22). Some common conditions, such as type 2 diabetes and hypertension, can usually be treated with low-cost first-line agents. However, our study showed that other conditions require costly treatments, namely hyperlipidemia, treated with a daily statin for the patient’s lifetime; chronic asthma, treated indefinitely with inhalers; and contraceptive management, requiring oral or injected contraceptive medication for years. The pattern in the most common comorbidities of our patient population is similar to the pattern found in a 2019 cross-sectional study of patients (n = 150) at 2 community health centers in Georgia: endocrine and metabolic disorders (86.0% vs 64.3% in our study), circulatory system diseases (79.3% vs 48.8% in our study), and mental disorders (25.3% vs 20.9% in our study) (1).

Our patients’ location of residence also validates existing literature on neighborhood-based disparities in medical access. One 2012 study described a lack of community pharmacies in low-income communities, segregated Black communities, and federally designated Medically Underserved Areas in Chicago (23). Another 2012 study found that pharmacies in low-income neighborhoods in New York City had significantly higher odds of having out-of-stock medications than pharmacies in higher-income neighborhoods (odds ratio = 1.24; 95% CI, 1.02–1.52) (24). A third study, in 2015, found increased medication prices and decreased access to home delivery services in socioeconomically disadvantaged neighborhoods in Tennessee (25). In our contemporary study, we observed a similar lack of accessible pharmacies in the low-income neighborhoods where our patients reside. As hundreds of retail pharmacies nationwide closed in 2023 due to increasing competition, opioid lawsuits, and other forces, pharmacy deserts are likely to increase in number (26).

Health insurance would help our patients receive partial or full coverage for prescription medications. However, in our patient population, many cannot afford private health insurance, many are not provided health insurance by their employers, and many do not qualify for low-income health insurance through Husky Health (Connecticut’s Medicaid). These factors account for the health insurance coverage gap described in the literature (27). We speculate that some patients at Haven — with no insurance, low health literacy, and language barriers (87% of our patients speak primarily Spanish) — may never obtain their medications or resort to paying retail price.

Haven’s policy is to provide every patient with the medication they need while doing its best to keep within an annual pharmacy budget of $10,000. To accomplish this goal, Haven seeks financial support from external programs to cover medication costs for patients at retail pharmacies. In particular, during the study period, the clinic received a 1-time $2,500 donation and up to $2,100 in monthly cash aid debit cards from the GoodRx Helps Medication Assistance Program (28). These funds were distributed directly to patients for medication purchases.

The clinic also applied to patient assistance programs on behalf of patients, such as the program administered by the Boehringer Ingelheim Cares Foundation (29) that provides free sodium-glucose cotransporter-2 inhibitors to patients with type 2 diabetes, and the program administered by the US Department of Health and Human Services that provides free access to daily oral HIV pre-exposure prophylaxis medication for people at risk of acquiring HIV. Although three 120-day rifampin medications (4-month rifampin monotherapy) were prescribed and paid for at $128.94 each during our study period, the Connecticut Department of Public Health now provides rifampin at no cost through a tuberculosis control program it implemented (30). In 2022 and 2023, Haven treated at least 12 patients annually with rifampin for latent tuberculosis through this program.

Because of the positive effect of patient assistance programs on medication access and affordability, Haven identified a list of patient assistance programs in which patients can be enrolled. The most frequently prescribed expensive medication in our study — fluticasone propionate/salmeterol (an inhaler prescribed to treat asthma) — was offered by GSK’s prescription assistance program as Advair Diskus/HFA. However, this program was not widely used by Haven’s patients because they often needed medication urgently and could not wait for program approval, which often took longer than anticipated and sometimes ended with a rejection or request for further documentation. Another common reason for not using GSK’s program was that patients preferred a newer inhaler with the same ingredients — AirDuo RespiClick. An additional hurdle associated with prescription assistance programs is that they often discontinue medications without much advance notice. For example, in summer 2023, GSK removed Advair Diskus/HFA from the list of medications available in its program (31), and AstraZeneca announced that Pulmicort Flexhaler (budesonide) and Symbicort (budesonide/formoterol) — also inhalers to treat asthma — would be phased out by the end of 2023 (32).

Currently, only 1 retail pharmacy in the New Haven area accepts payment over the telephone from the Haven pharmacy, which is likely the reason it is the most common pharmacy used by Haven’s patients. Paying for medications over the telephone on behalf of patients increases the speed, convenience, and likelihood of patients obtaining their prescription medication. Retail pharmacies offer some of the most affordable medication prices; however, some patients lack transportation to go to one of these pharmacies. Many pharmacies have declined accepting telephone payments because of company policy and to prevent fraud, but expanding the number of pharmacies where third parties can pay for patient medications via telephone would increase the options Haven can provide to patients to alleviate their financial burden. Additionally, expanding Haven’s options to deliver medications directly to patient homes would benefit patients. So far, these home delivery services have not been widely used at Haven because these medications tend to be more expensive and delivery can take 7 to 10 business days. Furthermore, not all of Haven’s patients have an address where they can receive mailed medications securely.

### Limitations

Our study has several limitations. First, our study is a single-center retrospective review and should be followed by a multicenter study that spans multiple states to capture data on differences in financial burden related to Medicaid expansion. Second, our study did not account for internal decisions made by health care providers to choose less expensive, alternative medications due to price, such as prescribing an AirDuo RespiClick (fluticasone propionate/salmeterol) asthma inhaler over the more expensive Pulmicort Flexhaler (budesonide) inhaler. Thus, the financial and medical effect of these decisions on patients may be greater than captured in our study. Lastly, our database did not include information on the use of prescription assistance programs and the associated cost savings. The Haven pharmacy department hopes to incorporate this into its future workflow.

### Conclusion

This study from the Haven free clinic demonstrates that patients without health insurance have a financial burden resulting from having to buy prescription medications for common chronic medical conditions, including hyperlipidemia and asthma. Although Haven is often able to provide patients with affordable treatment through the efforts of 24 student volunteers and codirectors who research and apply for medication assistance programs, seek the lowest-priced options, and many times even deliver medications to patients’ homes, student-run clinics cannot be easily scaled to all 7.7 million uninsured people in the US (33). Future research should seek ways to make medications, especially medications considered expensive (>$20), affordable. Removing financial barriers would improve medication adherence among patients that lack health insurance and may contribute to improved overall health.
