# Erratum, Volume 21, May 16 Release

**DOI:** 10.5888/pcd21.230277e

**Published:** 2024-06-13

The caption for Figure 1 in the article “The Cost of Medications at a Student-Run Free Clinic in New Haven, Connecticut, 2021–2023,” by See et al, was revised to reflect the appropriate attributions: “Location of neighborhoods in New Haven, Connecticut. A large proportion (62%) of Haven’s patients live in either the Hill or Fair Haven, 3 to 6 miles from Amity, which has the only retail pharmacy in New Haven that accepts payment over the telephone. Map tiles by Stamen Design, under CC BY 4.0 (15). Data by OpenStreetMap, under ODbL, adapted by the Yale MacMillan Center, which added static neighborhood labels to the image, and used with permission. In addition, 2 red circles were added to highlight low-income neighborhoods (Fair Haven and Hill), and a purple circle was added to highlight a high-income neighborhood (Amity).”

The article was updated on May 29, 2024, and is available here: https://www.cdc.gov/pcd/issues/2024/23_0277.htm. We regret any confusion this error may have caused.

